# Receptor for detection of a Type II sex pheromone in the winter moth *Operophtera brumata*

**DOI:** 10.1038/srep18576

**Published:** 2016-01-05

**Authors:** Dan-Dan Zhang, Hong-Lei Wang, Anna Schultze, Heidrun Froß, Wittko Francke, Jürgen Krieger, Christer Löfstedt

**Affiliations:** 1 Department of Biology, Lund University, SE-223 62 Lund, Sweden; 2 Institute of Physiology, University of Hohenheim, Stuttgart, Germany; 3 Institute of Organic Chemistry, University of Hamburg, Hamburg, Germany; 4 Department of Animal Physiology, MLU Halle-Wittenberg, Halle, Germany

## Abstract

How signal diversity evolves under stabilizing selection in a pheromone-based mate recognition system is a conundrum. Female moths produce two major types of sex pheromones, i.e., long-chain acetates, alcohols and aldehydes (Type I) and polyenic hydrocarbons and epoxides (Type II), along different biosynthetic pathways. Little is known on how male pheromone receptor (PR) genes evolved to perceive the different pheromones. We report the identification of the first PR tuned to Type II pheromones, namely ObruOR1 from the winter moth, *Operophtera brumata* (Geometridae). ObruOR1 clusters together with previously ligand-unknown orthologues in the PR subfamily for the ancestral Type I pheromones, suggesting that *O. brumata* did not evolve a new type of PR to match the novel Type II signal but recruited receptors within an existing PR subfamily. AsegOR3, the ObruOR1 orthologue previously cloned from the noctuid *Agrotis segetum* that has Type I acetate pheromone components, responded significantly to another Type II hydrocarbon, suggesting that a common ancestor with Type I pheromones had receptors for both types of pheromones, a preadaptation for detection of Type II sex pheromone.

Chemical signalling and perception is an important and widespread way of communication for living beings^1^. How signal diversity evolves under strong stabilizing selection against deviant signals and responses remains a conundrum^1^,^2^,^3^,^4^,^5^,^6^,^7^,^8^. Normally the genes controlling signal production and recognition are different and unlinked^1^,^2^, only with rare exceptions such as the *Drosophila desat1* gene that exerts the control on both sides^9^. Previous studies on how signal diversity can evolve provide us with insights from the signal perspective^3^,^4^,^10^,^11^,^12^,^13^,^14^. A recent study in jewel wasps shed light on signal evolution, indicating that the new pheromone compounds could arise through a simple mutation, and that they could persist in a sender population without being selected by a pre-existing preference^7^. In the perspective of perception, however, less is known about how the specificity of the receivers’ response could coevolve with the advertisement signals.

As a case of an efficient, species-specific and well-studied chemical communication system, moth sex pheromone communication has long been a model for the understanding of signaller-receiver evolution. According to their chemical characteristics, the vast majority of lepidopteran sex pheromones are straight chain, even numbered acetates, aldehydes, and alcohols with 10 to 18 carbon atoms. These so called Type I pheromone components make up the pheromones of around 75% of all moth species for which pheromones have been identified^15^. Type I pheromones evolved in the early radiation of the Lepidoptera^16^,^17^. In a few advanced superfamilies, mainly the Geometroidea and Noctuoidea, Type II pheromones have evolved. Type II pheromones consist of polyunsaturated C_17_-C_23_ straight-chain, mostly uneven-numbered, skipped conjugated polyenic hydrocarbons and the corresponding epoxide derivatives. This type of compounds comprises about 15% of the identified lepidopteran pheromones^15^,^18^,^19^. The two major types of sex pheromones are produced through distinct pathways, including differences in the biosynthetic sites, the substrates and enzymes involved, as well as the endocrine regulation mechanism, but still serve the same role in moth mate recognition and attraction^20^,^21^,^22^,^23^.

In male response to the conspecific sex pheromone, specialized sex pheromone receptors (PRs), expressed in olfactory sensory neurons (OSNs) on the male antennae, play a crucial role^24^. Genes encoding PRs are being isolated from an increasing number of lepidopteran species, but relatively few have been functionally characterized^25^, and references therein. The characterized lepidopteran PRs comprise a particular subfamily of insect olfactory receptors (ORs)^26^. To date, all the characterized PRs are receptors for Type I pheromones; no PRs responsive to Type II pheromones have been found. Thus, it is unknown how the transition from detection of ancestral Type I pheromones to Type II pheromones occurred. Did moths evolve a novel type of receptors to match the novel pheromone components or were existing receptors recruited and adapted to match the new signal?

The winter moth, *Operophtera brumata* (Geometridae) uses a C_19_ tetraene, (3*Z*,6*Z*,9*Z*)-1,3,6,9-nonadecatetraene (1,3Z,6Z,9Z-19:H), as a single-component sex pheromone^27^,^28^. This compound is a typical representative of the Type II pheromones. In addition, three polyene analogues, (3*Z*,6*Z*,9*Z*)-3,6,9-nonadecatriene (3Z,6Z,9Z-19:H), (3*Z*,6*Z*,9*Z*)-3,6,9-henicosatriene (3Z,6Z,9Z-21:H) and (3*Z*,6*Z*,9*Z*)-1,3,6,9-henicosatetraene (1,3Z,6Z,9Z-21:H), were found in the female pheromone gland, biosynthetically derived along similar pathways^29^. In the present study, we report the first identification of a receptor for a Type II pheromone component. Based on receptor gene characterization, functional studies and phylogenetic analysis we suggest that the receptor for Type II polyene pheromones was recruited from a cluster within the existing PR gene family with previously unknown ligand sensitivities.

## Results

### Morphology and sexual dimorphism of the antennal olfactory apparatus

**Figure 1:**
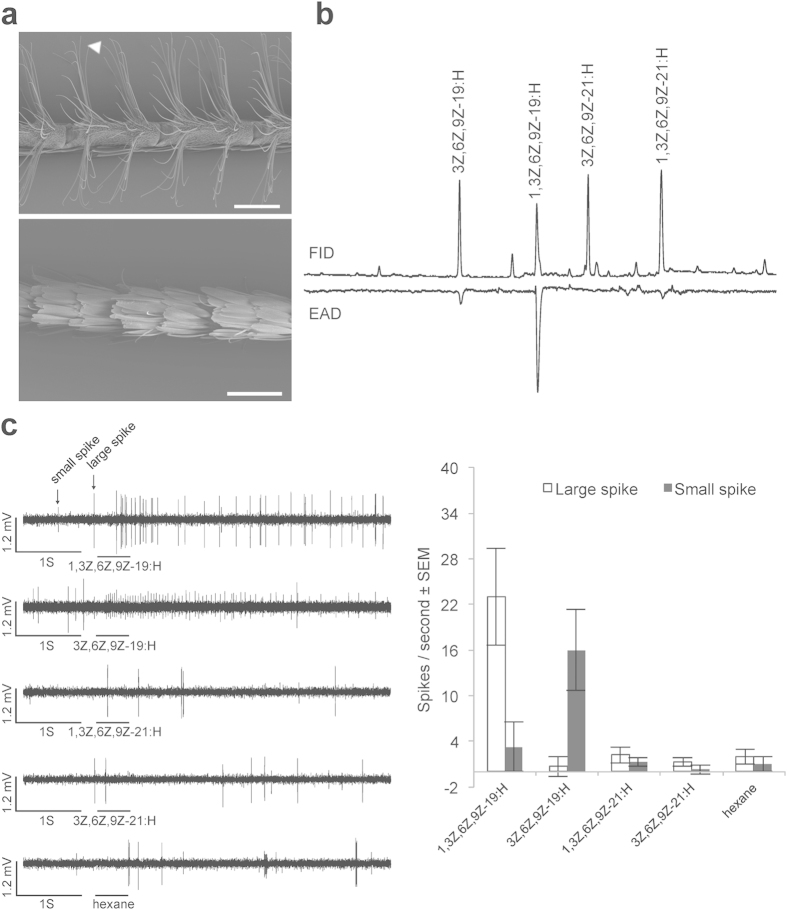
The sexual dimorphism of *O. brumata* antennae and the electrophysiological responses to the sex pheromone and polyene analogues. (**a**) SEM pictures of male antennae (upper) and female antennae (lower) showing the sexual dimorphism in the chemosensilla. The arrowhead indicates single long sensilla trichodea. Scale bar, 200 μm. (**b**) GC-EAD responses of male antennae to the synthetic sex pheromone and polyene analogues. (**c**) Responses of single sensilla trichodea on male antennae to the synthetic sex pheromone and polyene analogues at 10 μg doses.

We first examined the sexual dimorphism of the *O. brumata* antennae by using scanning electron microscopy (SEM). There are abundant sensilla trichodea distributed on the ventral and lateral sides across the whole male antennae, grouping into two clusters at both ends of each segment (Fig. 1a). This type of sensilla was proven to be involved in sex pheromone reception in various moth species^30^. In the female antennae, however, the sensilla are much less abundant and covered by scales, thus not as apparent as on the male antennae (Fig. 1a).

### Responses of male *O. brumata* antennae to the pheromone component and structural analogues

We tested the response of the male antennae to the tetraene pheromone and three polyene analogues by gas chromatography with electroantennographic detection (GC-EAD). The male antennae showed the strongest response to the C_19_ pheromone 1,3Z,6Z,9Z-19:H, and the second largest response to the C_19_ triene analogue, 3Z,6Z,9Z-19:H, a weak response to the bis-homolog tetraene 1,3Z,6Z,9Z-21:H, and no response to the bis-homolog triene 3Z,6Z,9Z-21:H (Fig. 1b).

### Responses from single sensilla

We subsequently tested the response of the single sensilla trichodea using the same set of compounds. For all the tested sensilla trichodea we observed a large-spike neuron responding to the tetraene pheromone, 1,3Z,6Z,9Z-19:H, and a small-spike neuron responding to the triene analogue 3Z,6Z,9Z-19:H, whereas the other two analogues elicited no response (Fig. 1c). In addition, increased spike frequencies were observed when the two antennal-active stimuli were applied at a 10x higher dose (data not shown).

### Candidate genes of PR and the co-receptor

We next aimed to identify the gene encoding the receptor tuned to the pheromonal tetraene. Based on the sequences of the previously functionally identified moth PRs, we designed degenerate primers and PCR amplified the candidate cDNA fragments from male antennae. We found three partial sequences and obtained one full-length sequence by 5′ and 3′-RACE PCR. We verified the gene by full-length amplification and sequencing, and termed it *ObruOR1* (Genbank accession number KJ592711). *ObruOR1* encodes a protein of 430 amino acids, sharing 58% identity with the candidate PR HvirOR11 in *Heliothis virescens* that belongs to the Noctuidae family^31^. The full-length of the other two partial sequences could not be obtained in the present study, probably because of the low abundance of the transcripts.

In addition, we cloned the olfactory co-receptor gene from *O. brumata*, which encodes a predicted protein of 472 amino acids and was termed *Obru\Orco* (Genbank accession number KJ592712) according to the unified nomenclature system for the insect olfactory co-receptor^32^. Obru\Orco shares 65%, 83%, and 88% amino acid identity with Orcos in *Drosophila melanogaster, Bombyx mori,* and *H. virescens*, respectively^33^,^34^,^35^.

### Male-biased expression of *ObruOR1*

**Figure 2:**
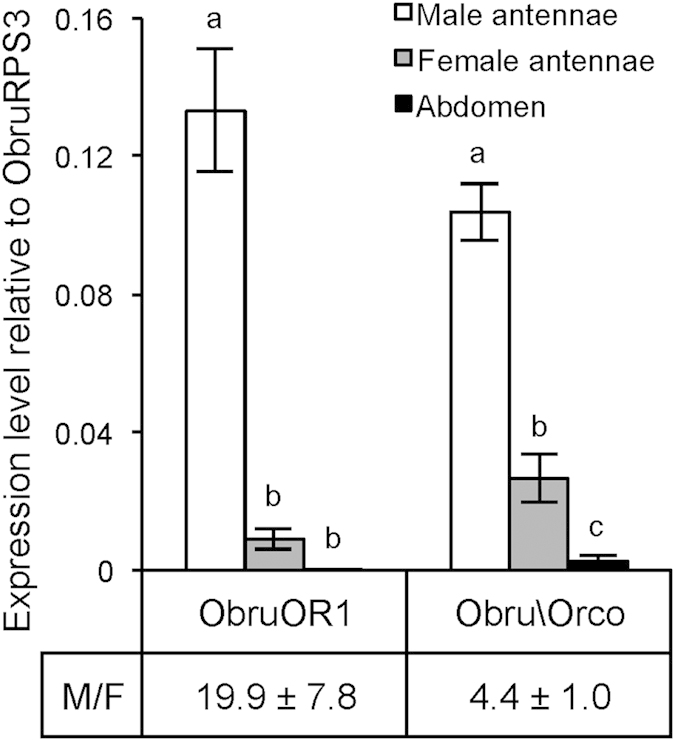
The expression levels of *ObruOR1* and *Obru\Orco* genes. The expression levels (Mean ± SE) of *ObruOR1* and *Obru\Orco* relative to the reference gene *ObruRPS3* in male and female antennae as determined by qPCR. Abdominal tissue is included as a control. Bars with different letters indicate expression levels significantly different at p < 0.01 (one-way ANOVA followed by a Tukey’s test, N = 3). Ratios of male to female expression (M/F) are presented below the bars.

Using qPCR, we compared the expression levels of *ObruOR1* and *Obru\Orco* genes in male and female antennae as well as in abdomens (Fig. 2). The expression level of *ObruOR1* was significantly higher in male antennae than in female antennae (19.9 ± 7.8-fold), suggesting its role in the detection of female produced sex pheromones by male individuals, whereas the gene showed very low expression in abdominal tissue. Transcripts of *Obru\Orco* were also more abundant in male antennae than in female antennae (4.4 ± 1.0-fold), probably due to the fact that the female antennae bear much fewer sensilla (Fig. 1a).

### Wide distribution of *ObruOR1* in the sensilla trichodea on male antennae

**Figure 3:**
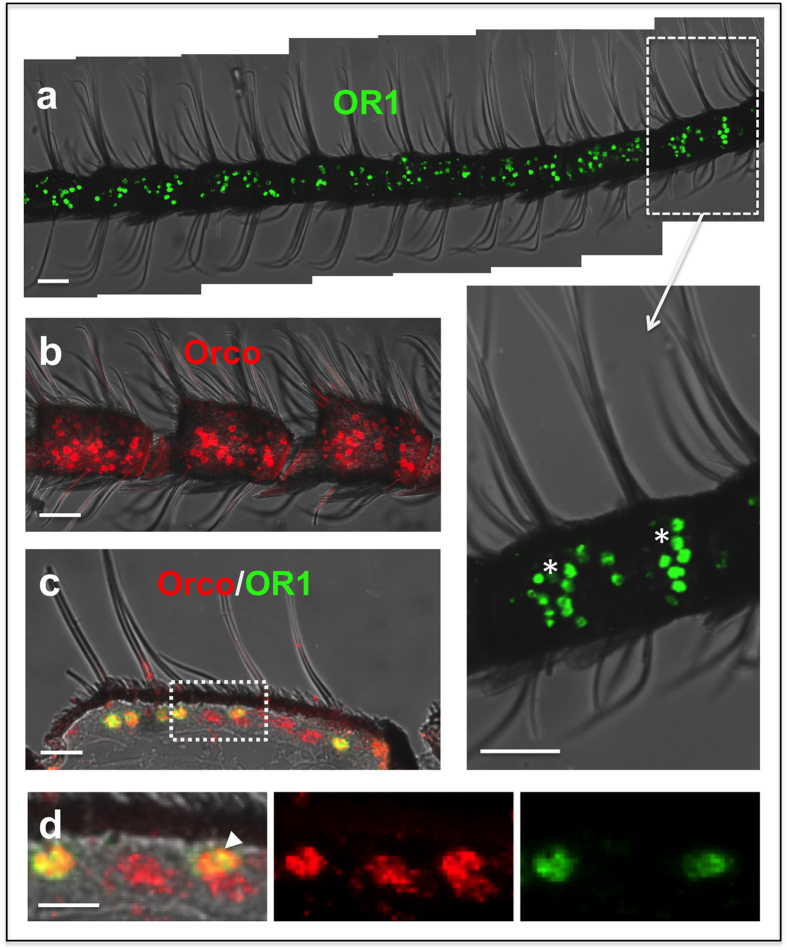
Fluorescence *in situ* hybridization of *ObruOR1* and *Obru\Orco* genes. Whole mount preparations were hybridized with biotin-labelled probes for *ObruOR1* as shown in green signal **(a)** and DIG-labelled probes for *Obru\Orco* as shown in red signal **(b)**. The asterisks in the higher magnification of a segment from **(a)** indicate the correspondence of the *ObruOR1* labelled cells with the two clusters of long sensilla trichodea. **(c,d)** Two-color FISH on longitudinal cryosection specimen of the antennae with *ObruOR1* and *Obru\Orco* visualized by green and red fluorescence, respectively. The boxed area from **(c)** is shown in higher magnification in **(d, left)**, where the yellow color indicates the overlay of the red **(d, middle)** and green fluorescence channels **(d, right)**, showing the co-expression of *ObruOR1* and *Obru\Orco* in the same cells. A pair of ORNs with one of the two adjacent cells expressing both *Obru\Orco* and *ObruOR1* and the other cell expressing *Obru\Orco* alone was indicated by arrowhead. Scale bars: 50 μm in **a** and **b**, 20 μm in **c**, 10 μm in **d**.

To visualize the expression pattern of *Obru\Orco* and *ObruOR1* in male antennae, we performed whole mount fluorescence *in situ* hybridization (FISH) with the digoxigenin (DIG) and biotin labelled antisense RNA-probes for *Obru\Orco* and *ObruOR1* respectively. *ObruOR1* was extensively expressed across the whole antennae. The labelled cell bodies were observed in clusters at both ends of each segment, exactly corresponding to the positions where the clusters of long sensilla trichodea are localized (Fig. 3a). Compared to *ObruOR1, Obru\Orco* was more widely expressed and distributed more evenly in each segment, associated not only to the long sensilla trichodea, but also to other olfactory sensilla (Fig. 3b).

Furthermore, we observed the co-expression pattern of *Obru\Orco* and *ObruOR1* on the longitudinal sections of male antennae via double *in situ* hybridization, where the red *Obru\Orco* labelling and the green *ObruOR1* labelling overlapped and displayed as yellow signals (Fig. 3c). In addition, we found examples of the pairing of OSNs in the long sensilla trichodea, where one of the two adjacent cells displayed a merged signal in yellow, indicating the presence of both *Obru\Orco* and *ObruOR1*, whereas the other cell showed a red signal of *Obru\Orco* alone (Fig. 3d).

### The specific receptor for the Type II sex pheromone of *O. brumata*

**Figure 4:**
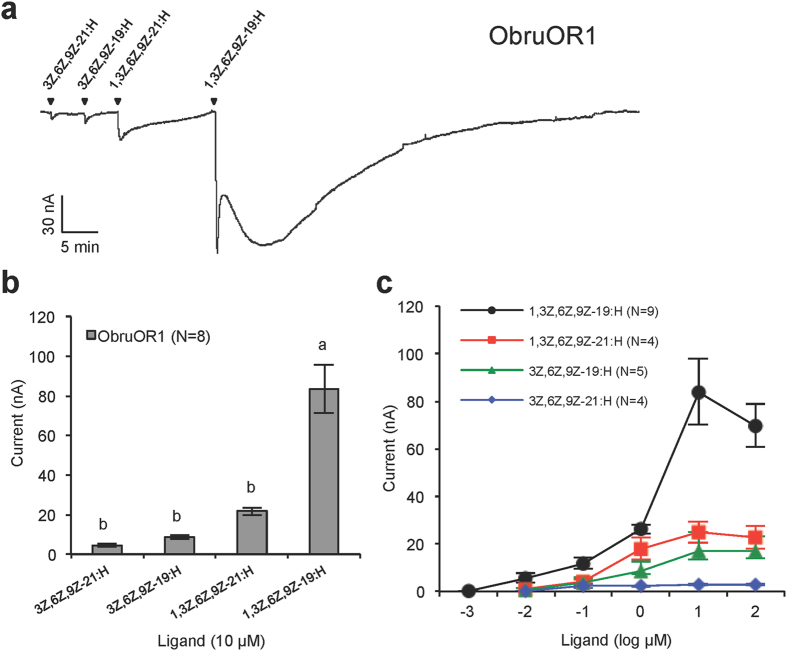
Functional assay of ObruOR1. **(a)** The current trace of a Xenopus oocyte co-injected with cRNAs encoding ObruOR1 and Obru\Orco upon successive exposures to 10 μM stimuli. Each chemical was applied for 10 s at the time point indicated by arrows. **(b)** The mean values ± SE of the stimulated currents in nano-Ampere (nA). Number of replicates is indicated in the parentheses. Different letters above bars represent values significantly different at p < 0.05 (one-way ANOVA followed by a Tukey’s test). **(c)** The responses of ObruOR1 at different doses of each stimulus. Numbers of replicates for each compound are indicated in the parentheses.

We tested the response profile of ObruOR1 by two-electrode voltage clamp (TEVC) recording on *Xenopus* oocytes co-expressing ObruOR1 and Obru\Orco. When challenged by the four polyenes found in the pheromone gland, the injected oocytes responded most strongly to the pheromonal tetraene 1,3Z,6Z,9Z-19:H, and only slightly to the analogues 1,3Z,6Z,9Z-21:H, 3Z,6Z,9Z-19:H, and 3Z,6Z,9Z-21:H (Fig. 4a,b). This ligand selectivity was further confirmed by a dose-response experiment. At all doses the response to the tetraene pheromone was stronger than those to the analogues. The threshold dose for 1,3Z,6Z,9Z-19:H was at 10 nM, ten times lower than for the other three compounds (Fig. 4c).

Notably, we found that after stimulating the ObruOR1 expressing oocytes with the pheromone 1,3Z,6Z,9Z-19:H, the inactivation of the inward current was much slower compared to what had been observed with the previously identified receptors for Type I pheromones. For the receptors of Type I pheromones, the recovery typically takes a few minutes^36^,^37^,^38^,^39^,^40^, however, it took approximately 40 min for ObruOR1 to get the inward current back to the baseline, even if we reduced the stimulation time from 20 s to 10 s or 5 s.

### Phylogenetic analysis of moth pheromone receptors

**Figure 5:**
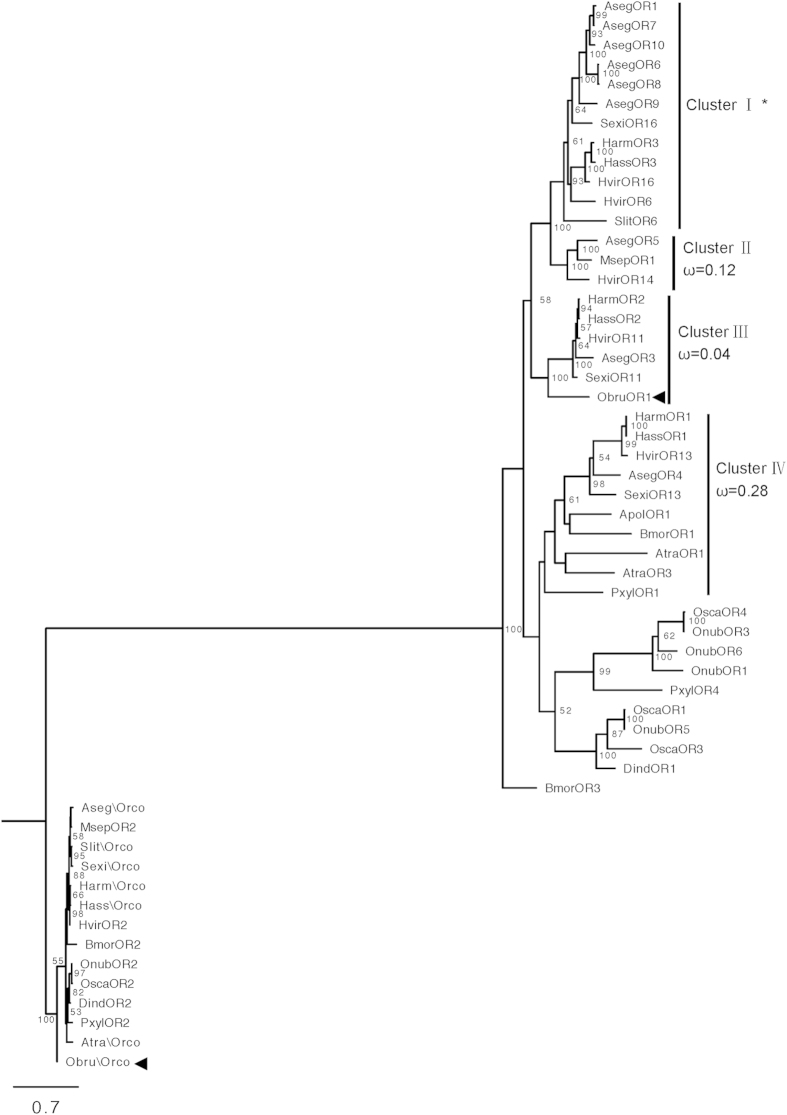
The phylogeny of lepidopteran PRs. The Maximum Likelihood tree with functionally identified lepidopteran PR sequences and ObruOR1 orthologues. The tree is rooted with Orco lineage. Percentage bootstrap support (100 replicates) values over 50 are shown at corresponding nodes. ObruOR1 and Obru\Orco are indicated with arrowheads. The ω values (ratio of nonsynonymous to synonymous substitutions, dN/dS) are shown in the tree. *Orthologous Cluster II, III and IV have a uniform ω rate for all branches, whereas Cluster I has various ω rates for all branches within the lineage.

We examined the phylogeny of lepidopteran PRs, including ObruOR1 and it´s ligand-unknown orthologues (Fig. 5). Following previous studies^25^,^40^, we recognize four apparent orthologous clusters containing receptors from the most advanced lepidopteran families. ObruOR1 is not separated from previously reported PRs, but clusters with bootstrap support 100 within Cluster III. Except for ObruOR1, Cluster III only contains candidate PRs of noctuid species. It consists of a group of ligand-unknown PRs and is under strong purifying selective pressure, indicated by the previously reported ω value (or dN/dS, the ratio of nonsynonymous to synonymous substitutions) significantly less than 1^40^. We re-calculated the ω value of this cluster after the addition of ObruOR1. By comparing the log likelihoods (lnL) of model M0 (assuming one ω ratio for all branches) and model M1 (assuming one ω ratio for each branch), we found that the one ratio model (M0) could not be rejected (P>0.01). Thus, all branches in Cluster III share a normalized ω ratio (ω = 0.04). The low ω ratio suggests that this cluster is under strong purifying selection (Fig. 5, Table S1), which may result in a conserved ligand profile of the orthologous receptors.

### Functional assay of a noctuid orthologue in Cluster III

**Figure 6:**
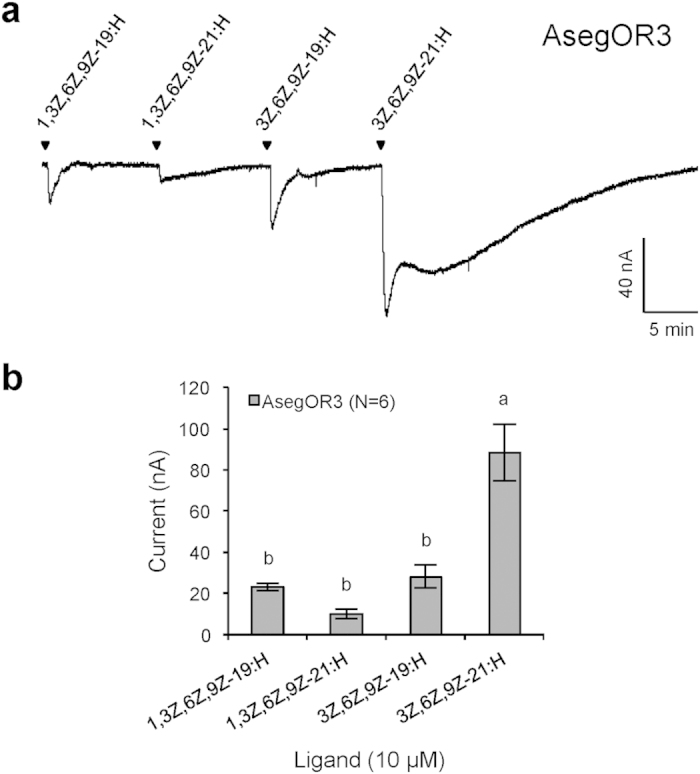
Functional assay of an ObruOR1 orthologue–AsegOR3. **(a)** The current trace of a Xenopus oocyte co-injected with cRNAs encoding AsegOR3 and Aseg\Orco upon successive exposures to 10 μM stimuli. Each chemical was applied for 10 s at the time point indicated by arrows. **(b)** The mean values ± SE of the stimulated currents in nano-Ampere (nA). Number of replicates is indicated in the parentheses. Different letters above bars represent values significantly different at p < 0.05 (one-way ANOVA followed by a Tukey’s test).

Active ligands for PRs in Cluster III have not been previously reported. To test the above hypothesis of a conserved ligand profile within this cluster, we co-expressed AsegOR3, a noctuid orthologue that showed very weak responses to Type I pheromone components in our previous study^40^, with the corresponding co-receptor Aseg\Orco from the turnip moth *Agrotis segetum*, and tested it against the four polyenes that we used for ObruOR1. Indeed, the orthologous receptor responded strongly to the triene 3Z,6Z,9Z-21:H, but less so to 3Z,6Z,9Z-19:H, 1,3Z,6Z,9Z-21:H, and the *O. brumata* pheromone tetraene 1,3Z,6Z,9Z-19:H (Fig. 6).

## Discussion

Males and females of the winter moth *O. brumata* show clear sexual dimorphism in their antennal morphology. We showed that a receptor neuron in sensilla trichodea on the male antennae, responded strongly to the pheromone tetraene 1,3Z,6Z,9Z-19:H. These neurons expressed the pheromone receptor gene ObruOR1, which responded specifically to the tetraene when tested in the *Xenopus* oocyte assay. This is the first identification of a receptor for a lepidopteran Type II pheromone.

ObruOR1 resembles receptors for Type I pheromones in most aspects. It shows male-biased expression comparing antennae from males and females, as most of the receptors for Type I pheromones^25^, and references therein. The expression is confined to long sensilla trichodea, a type of sensilla relevant to the detection of sex pheromones in moths^30^. The single-sensillum recording (SSR) results demonstrate that in the long sensilla trichodea, the large-spike neuron is responsive to the tetraene pheromone, 1,3Z,6Z,9Z-19:H, and the neighbouring small-spike neuron is responsive to the triene analogue, 3Z,6Z,9Z-19:H (Fig. 1c). We, therefore, suggest that ObruOR1 is assigned to the large-spike neuron in the long sensilla trichodea and that another unidentified receptor is expressed in the adjacent small-spike neuron responding to 3Z,6Z,9Z-19:H.

Compared to previously identified PRs, the ObruOR1 showed a much slower inactivation of the inward current to the target ligand 1,3Z,6Z,9Z-19:H in the TEVC recording (Fig. 4a). The immediate strong response was followed by an unusual slower secondary response. Notably the same pattern was observed when stimulating AsegOR3 with 3Z,6Z,9Z-21:H, the most active ligand on this receptor. We can only speculate on the explanation for these patterns. The slow inactivation process might imply a much higher affinity between the polyene and the receptor in the heterologous system than that between Type I pheromones and corresponding receptors. Under natural conditions, other related proteins such as pheromone degrading enzymes or sensory neuron membrane proteins may be involved to facilitate the fast release of the bound polyenes from the receptors and thus accelerate the inactivation process and maintain an effective signal transduction, whereas in the oocytes such proteins are not present.

The winter moth pheromone receptor ObruOR1 shares sequence identity with the receptors for Type I pheromones and nests within the orthologous Cluster III together with noctuid receptors in the phylogenetic tree. Previously, no active ligands for the ObruOR1 orthologues in Cluster III have been reported. Although they have been assumed to be relevant to the reception of structurally similar pheromones, behavioural antagonists, or the degradation products of the major sex pheromone component^41^,^42^, these assumptions have not received any support by functional data. We noticed that Cluster III is under strong purifying selection (Fig. 5). This is an indication of conserved ligand profiles, which suggested to us that these orthologues might also be responsive to the Type II pheromone compounds. Indeed, we found that AsegOR3, one of the orthologues, did respond to the tested polyenes which lends support to this hypothesis. To our knowledge, the noctuid species hosting the PR orthologues in Cluster III do not use polyenes as sex pheromones. Thus the fact that one of these PRs is responsive to polyene compounds is of great interest, and the behavioural significance of this finding as well as the active ligands of the other orthologues await further studies.

Our results demonstrate that evolution of the novel Type II pheromones in Lepidoptera does not require the evolution of entirely novel receptors. Instead, members of the existing pheromone receptor subfamily can be recruited for detection of the new ligands. This is different to the production of the two types of pheromones in female moths, where distinct pathways and enzymes are involved^21^,^22^. Type II pheromones mainly appear in the advanced moth superfamilies Geometroidea and Noctuoidea^17^ and we have not found any ObruOR1 orthologues outside Geometridae and Noctuidae among available sequences of PR candidate genes. We suggest that Cluster III might have evolved in a common ancestor of the geometroid and noctuoid lineages, but testing this hypothesis will require cloning and functional characterization of pheromone receptors in a number of additional phylogenetically relevant species. Interestingly, in the Pyraloidea superfamily some species use both Type I and Type II pheromone components^43^,^44^,^45^. The co-existence of the two types of pheromone components in the same species is intriguing and invites studies of the evolution of receptors for Type II pheromone components in the pyraloids.

## Materials and Methods

### Ethics statement

The care and use of *Xenopus laevis* frogs in this study were approved by the Swedish Board of Agriculture, and the methods were carried out in accordance with the approved guidelines.

### Insect material

The insects used in this study were collected in early November of 2011–2013 in Lund from a small wooded area (55°42´ N, 13°12´ E) comprised of a mix of oak and chestnut trees.

### Chemicals

For the synthesis of 3Z,6Z,9Z-19:H and 3Z,6Z,9Z-21:H, the tosylate of (9*Z*,12*Z*,15*Z*)-octadeca-9,12,15-trienol^46^ was chain elongated with methyl magnesium iodide resp. propyl magnesium iodide employing cuprate chemistry^47^ and using the protocol of Molnár *et al*.^48^. The synthesis of 1,3Z,6Z,9Z-19:H and 1,3Z,6Z,9Z-21:H started from commercially available (Aldrich) 1-bromononane and 1-bromoundecane, following the iterative chain extension described by Zhu *et al*.^49^. Spectroscopic data of the four polyenes were in accord with those reported in the literature^50^,^51^,^52^.

### GC-EAD

An Agilent 7890 gas chromatograph equipped with a flame ionization detector (FID) and a capillary column of HP-INNOWax (30 m ×0.25 mm i.d., and 0.25 μm film thickness; J&W Scientific, USA) was used to monitor the physiological activities of the synthetic compounds. Male adults (N = 5), one to two day-old, were used in these experiments. After cutting off the tips, the antennae together with the head were mounted to a PRG-2 EAG (10X gain) probe (Syntech, Kirchzarten, Germany) by conductive gel (Blågel, Cefar, Malmö, Sweden). The GC inlet was set at 230 °C, and a split of the carrier gas (hydrogen) at the end of the column allowed a 1:1 split of the GC effluent to the FID and the antennal preparation. Charcoal-filtered and humidified air passed over the antennal preparation from a glass tube outlet, which was connected to the GC transfer line heated at 255 °C. The oven temperature was programmed from 80 °C for 1 min, at a rate of 10 °C/min to 210 °C, hold for 10 min and then to 230 °C at 10 °C/min, hold for another 10 min. Data were analysed with the software GC-EAD Pro Version 4.1 (Syntech, Kirchzarten, Germany).

### SSR

For the antennal preparation, a male *O. brumata* was placed in a 200 μL pipette tip fastened by dental wax on a glass slide. The antennae were carefully pulled out from the tip cut opening and fixed on a cover slip using double sided sticky tape. The recording was performed using sharpened tungsten microelectrodes, of which the reference electrode was inserted through the eye, and the recording electrode was inserted in the base of the long sensillum trichodea. The antennae were flushed with a charcoal-filtered and humidified airflow at a rate of 0.5 mL/s through a glass tube (i.d. 5.9 mm). The outlet of the tube was approximately 25 mm away from the antennae. Each tested polyene was diluted in hexane to two working concentrations, 1 μg/μL and 10 μg/μL, respectively. Ten microliters of a one-component solution were loaded on a piece of filter paper (1 × 2 cm) and inserted into a Pasteur pipette. Four to ten replicates were recorded for different tested compounds. The stimulus was introduced into the airflow at a rate of 3 mL/s for 500 ms by a stimulus controller CS-02 (Syntech). The responses were recorded and analysed with Autospike software v3.9 (Syntech).

### RNA preparation and first strand cDNA synthesis

For each RNA sample, approximately 40 pairs of antennae were dissected from male or female adults. The dissected material was separately homogenized on ice in Trizol reagent (Invitrogen, Carlsbad, CA, USA) using a tissue tearor. Total RNA isolation was performed immediately after homogenization according to the manufacturer’s instructions. In the qPCR experiment, the total RNA was treated with TURBO RNase free DNase (Life technologies), followed by acid phenol: chloroform (Life technologies) purification. Non-RT control PCR was performed to verify the absence of genomic DNA contamination. First-strand cDNAs were synthesized in a 20 μL reaction mixture containing 150 ng total RNA and 1 μL of oligo-dT primers by ThermoScript RT-PCR System (Invitrogen, Carlsbad, CA, USA).

### Gene cloning

The cloning strategy was as previously described^40^,^53^. Briefly, degenerate primers were designed based on the lepidopteran pheromone receptor and Orco sequences (Table S3). To amplify the partial cDNA sequences, two rounds of PCR were performed. Several pairs of primers for RACE PCR were subsequently designed based on the cDNA fragment sequences (Table S3). According to the assembled full-length cDNA sequences, primers containing restriction sites were designed for amplifying the full-length cDNAs, which were subcloned into the pCS2+ vectors (Table S3). All the sequences were verified with a capillary 3130xL Genetic Analyzer (Applied Biosystems, Carlsbad, CA, USA) and analysed with Vector NTI Advance 10 software (Invitrogen, Carlsbad, CA, USA).

### Quantitative PCR

The relative expression levels of *ObruOR1* and *Obru\Orco* transcripts in male and female antennae were compared by qPCR on a Stratagene Mx3005P Real-Time PCR System (Agilent technologies), with RPS3 as the reference gene^26^,^38^,^54^ and the abdomen as the control tissue. Primers used for qPCR are shown in Table S3. Prior to qPCR, we performed RT-PCR and confirmed that RPS3 is expressed at similar level in different tissues (Figure S1). The primer efficiencies were validated by standard curves with 10X serial dilutions of the cDNA templates. Three biological replicates were performed in separate plates, with three technical replicates on each plate. The reaction was carried out in a 20 μL system containing 1 μL cDNA template, 10 μL Power SYBR Green PCR 2X Master Mix (Life technologies), and 0.3 μM of each primer. The thermal cycling parameters consisted of an initial denaturation step at 95 °C for 10 min, followed by 40 cycles of 95 °C for 15 s and 60 °C for 1 min. No-template controls were run in parallel for each primer pair. A subsequent melting curve analysis was performed to ensure the primer specificity. Gel electrophoresis analysis was carried out after amplification to confirm the amplicon size. The expression level of each gene relative to RPS3 was calculated by the comparative CT method^55^.

### Fluorescence *in situ* hybridization

Biotin- or digoxigenin-labelled antisense riboprobes for *ObruOR1* and *Obru\Orco* were transcribed from linearized recombinant pCS2+ plasmids using the T7 RNA transcription system (Roche). *In situ* hybridization with either a single probe or double probes was performed following the previously described protocols^31^,^34^,^56^. Briefly, digoxigenin-labelled probes were detected by anti-digoxigenin AP-conjugated antibodies in combination with the substrate HNPP/Fast Red (Fluorescent detection Set; Roche), and biotin-labelled probes were detected by streptavidin horseradish peroxidase conjugates in combination with the substrate FITC-tyramides in a TSA kit (Perkin Elmer, Boston, MA, USA). A sense probe was included as control when *in situ* hybridization with single probe was performed (Figure S2). In addition, the two probes with distinct sequences (*ObruOR1* and *Obru\Orco*) worked as controls of each other. For cryosections specimen, antennae were first embedded in Tissue-Tek O.C.T. compound (Sakura Finetek Europe, Zoeterwoude, the Netherlands) and sliced by 10 μm thickness at −20 °C. The cryosections were then thaw mounted on slides for subsequent hybridization and washes. For whole mount specimen, antennae were cut into pieces of ca. 4–5 mm length and gently squeezed by fine forceps. After the hybridization and washes in 0.25 mL PCR tubes, the tissues were mounted in mowiol solution. The slides were visualized on a laser-scanning microscope (Zeiss LSM510 Meta, Oberkochen, Germany).

### Functional assay of candidate PRs

The oocyte microinjection and two-electrode voltage clamp recording were performed as previously described^40^. The oocytes were collected from adult female *X. laevis* (purchased from Xenopus Express France, Vernassal, Haute-Loire, France) and pretreated by 1.5 mg/mL collagenase (Sigma-Aldrich Co., St. Louis, MO, USA). cRNAs of *ObruOR1* and *Obru\Orco* were synthesized from the linearized recombinant pCS2+ plasmids with mMESSAGE mMACHINE Kit (Ambion), and co-injected into the oocytes in the amount of 50 ng of each cRNA per oocyte. The injected oocytes were incubated at 18 ± 1 °C for 3–5 days, then the whole-cell inward currents at the holding potential of −80 mV were recorded by the two-electrode voltage clamp coupled with a TEC-03BF amplifier (npi electronic GmbH, Tamm, Germany). Oocytes without cRNA injection were set as negative control. Stock solutions of tested compounds were prepared using dimethyl sulfoxide (DMSO) (Sigma-Aldrich Co., St. Louis, MO, USA), which were diluted to the indicated concentrations by Ringer’s buffer before use. A perfusion system was used to apply the tested compounds to the oocytes at a rate of 2 mL/min. After the stimulation, the cell chamber was extensively washed with Ringer’s buffer at a flow rate of 4 mL/min to recover the inward current to baseline. Ringer’s buffer containing 0.1% DMSO alone was used as solvent control. Data were collected and analysed by Cellworks software (npi electronic GmbH, Tamm, Germany).

### Phylogenetic construction and selective pressure analysis

The evolutionary history was inferred with the software MEGA 6 by using the Maximum Likelihood method based on the LG model^57^,^58^. The so far functionally characterized lepidopteran PRs were included, as well as ObruOR1 and it´s ligand-unknown orthologues. Initial tree for the heuristic search was obtained by applying the Neighbor-Joining method to a matrix of pairwise distances estimated using a JTT model. A discrete Gamma distribution was used to model evolutionary rate differences among sites (5 categories (+G, parameter ≈ 1.2)). The tree was rooted with the Orco lineage. Bootstrap analysis of 100 replicates was performed. The ω value was estimated by the maximum likelihood method^59^ using the Codeml program in the PAML 4.6 package^60^. Genbank accession numbers of all the PR protein sequences in the tree are presented in Table S2.

## Additional Information

**How to cite this article**: Zhang, D.-D. *et al.* Receptor for detection of a Type II sex pheromone in the winter moth *Operophtera brumata. Sci. Rep.* **6**, 18576; doi: 10.1038/srep18576 (2016).

## Supplementary Material

Supplementary Information
