# Supplementary material for: Receptor for detection of a Type II sex pheromone in the winter moth Operophtera brumata
Source: Sci Rep. 2016 Jan 5;6:18576. doi: 10.1038/srep18576 (PMC4700456; doi:10.1038/srep18576)
Supplement: Supplementary Information [file srep18576-s1.pdf]

Supporting Information

Receptor for detection of a Type II sex pheromone in the winter moth *Operophtera brumata*

Dan-Dan Zhang[1], Hong-Lei Wang[1], Anna Schultze[2], Heidrun Froß[2], Wittko Francke[3], Jürgen Krieger[2,4], Christer Löfstedt[1,*]

Author affiliations

[1] Department of Biology, Lund University, Sölvegatan 37, SE-223 62 Lund, Sweden.

[2] Institute of Physiology, University of Hohenheim, Stuttgart, Germany.

[3] Institute of Organic Chemistry, University of Hamburg, Hamburg, Germany.

[4] Department of Animal Physiology, MLU Halle-Wittenberg, Halle, Germany.

christer.lofstedt@biol.lu.se

Running title: Receptor for Type II moth pheromone

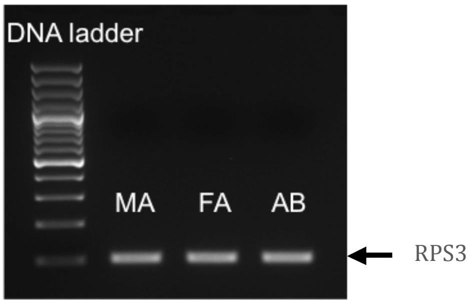

Figure S1. RT-PCR result confirming similar level of the reference RPS3 gene in different tissues. MA: male antennae; FA: female antennae; AB: abdomen.

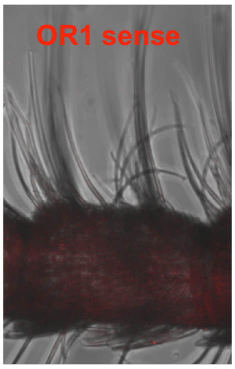

Figure S2. Sense probe control for in situ hybridization with single probe. The representative figure shows the image from whole mount preparation hybridized with DIG-labelled sense probe for *ObruOR1*.

Table S1. The selective pressures acting on PR orthologous clusters.

Cluster	n	InL M0	InL M1	df	*P* value
**I**	10	-6515	-6457	17	*P*<0.001
**II**	3	-3830	-3837	3	*P*>0.01
**III**	6	-8815	-8810	9	*P*>0.01
**IV**	10	-14666	-14680	17	*P*>0.01

Selective pressures acting on four orthologous clusters were tested by the branch-specific models in codeml (PAML 4.6 package). The log likelihoods (lnL) for branch models M0 (assuming one nonsynonymous to synonymous substitution rate for all branches) and M1 (assuming one nonsynonymous to synonymous substitution rate for each branch) were compared in likelihood ratio tests (LRT). *P* values were determined by comparison of the LRT statistics to a $\chi 2$ table using the relevant number for degrees of freedom. M0 model is a better fit for Clusters II-IV, and M1 model is a better fit for Cluster I. n = number of sequences; df = degrees of freedom.

Table S2. GenBank accession numbers of the PRs used in the phylogenetic tree.

PR protein name	Accession no.
Operophtera brumata	
ObruOR1	AJF20961
Agrotis segetum	
AsegOR1	AGS41441
AsegOR3	AGS41442
AsegOR4	AGS41443
AsegOR5	AGS41444
AsegOR6	AGS41445
AsegOR7	AGS41446
AsegOR8	AGS41447
AsegOR9	AGS41448
AsegOR10	AGS41449
Heliothis virescens	
HvirOR11	CAG38112.1
HvirOR13	CAG38114.1
HvirOR14	CAG38115.1
HvirOR16	CAG38117.1
Bombyx mori	
BmorOR1	BAD69584
BmorOR3	BAD89567
Plutella xylostella	
PxylOR1	BAG71420.1
PxylOR4	AGK43826.1
Mythimna separata	
MsepOR1	BAG71414.1
Diaphania indica	
DindOR1	BAG71417.1
Ostrinia scapulalis	
OscaOR1	BAH57975.1
OscaOR3	BAI66604.1
OscaOR4	BAI66605.1
Ostrinia nubilalis	
OnubOR1	ADB89178.1
OnubOR3	ADB89180.1
OnubOR5	ADB89182.1
OnubOR6	ADB89183.1
Antheraea polyphemus	
ApolOR1	CBH19582.1
Spodoptera littoralis	
SlitOR6	ACL81183.1
Amyelois transitella	
AtraOR1	AFP54146.1
AtraOR3	AFP54147.1
Helicoverpa armigera	
HarmOR1	ACS45304
HarmOR2	ACS45305

HarmOR3	ACS45306
Helicoverpa assulta	
HassOR1	ACS45307
HassOR2	ACS45308
HassOR3	ACS45309
Spodoptera exigua	
SexiOR11	AGH58120.1
SexiOR13	AGH58121.1
SexiOR16	AGH58122.1
**Orco lineage**	
Obru\Orco	AJF20962
Aseg\Orco	AGS41440
HvirOR2	CAD31851.1
BmorOR2	BAD69585
PxylOR2	BAG71421.2
MsepOR2	BAG71415.1
DindOR2	BAG71418.1
OscaOR2	BAH57973.1
OnubOR2	ADB89179.1
Slit\Orco	ABQ82137.1
Atra\Orco	AFP54145.1
Harm\Orco	ADQ13177
Hass\Orco	ADQ13178
Sexi\Orco	AAW52583.1

Table S3. Primer used in this article.

Primer	Primer sequences (5'→3')	
	**Degenerate primers for RT-PCR**	
**B1**	CARCARYTIATHCARYTITC	**S**
**B2a**	CAACAAYTNATHCAAATHTC	**S**
**B2b**	CAACAAYTNATHCAAATHAG	**S**
**C1**	TNCCDTGGGARTRYATGG	**S**
**C2**	TNCCDTGGGARKCYATGG	**S**
**C3**	TNCCDTGGGARAGYATGG	**S**
**D1**	ATNGANGCCATNGTIGTIACICC	**AS**
**D2**	NGYCATNGWYTGIACICC	**AS**
**D3**	ATCGCNGYCATNGAIGTIACICC	**AS**
**E1**	TAIGAIAWIGAIGTYTTYARDAT	**AS**
	**Primers for RACE PCR**	
**ObruOR1L**	TGAACTCCAAGAACCGGATGACTGC	**S**
**ObruOR1R**	ACGTCCACCATCCCGCCAGCAGT	**AS**
**Obru\OrcoL**	CGCCTTCAGTACACTTGGGTACCTGTG	**S**
**Obru\OrcoR**	TTCGCTTCTTCAGAGCCGTCGTACC	**AS**
	**Primers for full-length sequencing**	
**ObruOR1fl5**	*CGC*GGATCC**GCCACC**ATGGGGAGCCTTGACAATAAGGAC	**S**
**ObruOR1fl3**	*CCG*CTCGAGTTATTCAGCTCCAACTGTGCGAAG	**AS**
**Obru\Orco1fl5**	*CG*GAATTC**GCCACC**ATGATGACTAAGGTGAAATATCAGGGC	**S**
**Obru\Orco1fl3**	*CCG*CTCGAGTTATTTAAGTTGCACCAACACCATGAA	**AS**
	**Primers for reference gene cloning**	
**ObruRPS3L**	CGTGCNTGYTAYGGTGTNCT	**S**
**ObruRPS3R**	GTNACCARDATRTGRTCIGG	**AS**
	**Primers for qPCR**	
**ObruRPS3q5**	TGACCCGTGCAATGACTAC	**S**
**ObruRPS3q3**	CTGATCCCACGGCAACATAA	**AS**
**ObruOR1q5**	CACGGTGTACTTGTCCTGTATC	**S**
**ObruOR1q3**	AGCGTGTTCGTAAGTGGTATT	**AS**
**Obru\Orcoq5**	GTGCTTCTCTGGACACGTATAG	**S**
**Obru\Orcoq3**	CCCTCGGATGTCTATGTCTACT	**AS**

S: sense primer; **AS**: antisense primer. The underlined indicate restriction recognition sites, the italic indicates bases flanking the recognition sequences, and the bold indicate Kozak sequence.
